# Anaerobic bacteria in wastewater treatment plant

**DOI:** 10.1007/s00420-018-1307-6

**Published:** 2018-03-28

**Authors:** Marcin Cyprowski, Agata Stobnicka-Kupiec, Anna Ławniczek-Wałczyk, Aleksandra Bakal-Kijek, Małgorzata Gołofit-Szymczak, Rafał L. Górny

**Affiliations:** Central Institute for Labour Protection-National Research Institute, 16 Czerniakowska St., 00-701 Warsaw, Poland

**Keywords:** Wastewater treatment plant, Anaerobic bacteria, Exposure assessment, PCR, Size distribution

## Abstract

**Purpose:**

The objective of this study was to assess exposure to anaerobic bacteria released into air from sewage and sludge at workplaces from a wastewater treatment plant (WWTP).

**Methods:**

Samples of both sewage and sludge were collected at six sampling points and bioaerosol samples were additionally collected (with the use of a 6-stage Andersen impactor) at ten workplaces covering different stages of the technological process. Qualitative identification of all isolated strains was performed using the biochemical API 20A test. Additionally, the determination of *Clostridium* pathogens was carried out using 16S rRNA gene sequence analysis.

**Results:**

The average concentration of anaerobic bacteria in the sewage samples was 5.49 × 10^4^ CFU/mL (GSD = 85.4) and in sludge—1.42 × 10^6^ CFU/g (GSD = 5.1). In turn, the average airborne bacterial concentration was at the level of 50 CFU/m^3^ (GSD = 5.83) and the highest bacterial contamination (4.06 × 10^3^ CFU/m^3^) was found in winter at the bar screens. In total, 16 bacterial species were determined, from which the predominant strains belonged to *Actinomyces, Bifidobacterium, Clostridium, Propionibacterium* and *Peptostreptococcus* genera. The analysis revealed that mechanical treatment processes were responsible for a substantial emission of anaerobic bacteria into the air. In both the sewage and air samples, *Clostridium perfringens* pathogen was identified.

**Conclusions:**

Anaerobic bacteria were widely present both in the sewage and in the air at workplaces from the WWTP, especially when the technological process was performed in closed spaces. Anaerobic bacteria formed small aggregates with both wastewater droplets and dust particles of sewage sludge origin and as such may be responsible for adverse health outcomes in exposed workers.

## Introduction

Wastewater is always contaminated with different biological agents such as bacteria, viruses, protozoa, fungi, flatworms or roundworms (Sorber and Sagik 1980). Among them, pathogenic bacteria pose the most serious epidemiological risk. Wastewater can carry many opportunistic pathogens (e.g., *Enterobacter cloacae, Enterococcus faecalis, Escherichia coli, Klebsiella pneumoniae, Proteus vulgaris* or *Pseudomonas aeruginosa*), which can cause different systemic infections, especially among people with a weakened immune system. In wastewater can be also found obligate pathogens from *Salmonella* and *Shigella* genera or enteropathogenic strains of *Escherichia coli*, which are responsible for salomonellosis, shigellosis or gastroenteritis, respectively (Cyprowski et al. 2005; Gerardi and Zimmerman 2005).

From the oxygen demand standpoint, a majority of bacteria which may occur in this environment are aerobic ones. However, some of them can also survive having only temporary access to the oxygen (facultative anaerobes) or even without it (obligate anaerobes). A substantial part of the anaerobic bacteria is delivered to the wastewater treatment plant (WWTP) by the sewage network. In the recent study of Liu et al. (2015a), 18 species of *Longilinea, Georgenia, Desulforhabdus, Thauera, Desulfuromonas* and *Arcobacter* genera were identified in the sewerage system. Furthermore, anaerobic bacteria are an important element in the wastewater treatment processes. They are responsible for methane fermentation of sewage sludge, facilitating decomposition of macromolecular organic matter into simpler compounds. Among the bacterial genera involved in the anaerobic methane fermentation process are *Methanosarcina, Methanosaeta* (Van Lier et al. 2008; Zinder and Mah 1979) and *Clostridium* (Lisle et al. 2004; Wang et al. 2003). In treated effluent, there may be faecal bacteria of the genera *Bifidobacterium* and *Bacteroides* (Wery et al. 2010) as well as *Clostridium perfringens* (Ajonina et al. 2015).

During the wastewater treatment processes, bioaerosol is released into the air in the form of nuclei droplets, where the fine particles of water serve as carriers of microorganisms. At WWTPs, bacterial concentrations in the air usually range from 10^1^ to 10^4^ CFU/m^3^ (Korzeniewska 2011) and can adversely affect the health of sewage workers being responsible for respiratory, digestive tract, eye and skin infections (Cyprowski and Krajewski 2003). Despite the progress in research concerning physiological characteristics of anaerobic bacteria, the knowledge about their presence in working environments is still scarce. Limited data indicate that they may occur at all stages of wastewater treatment and their concentrations may be higher in winter (10^1^–10^4^ CFU/m^3^) than in summer (10^1^–10^2^ CFU/m^3^). However, the authors of such study did not usually present the detailed qualitative characteristics of anaerobic bacteria (Fracchia et al. 2006). Another study (Pillai et al. 1996) showed that sludge can be a source of bacteria of the genus *Clostridium*. These bacteria can be treated as microbial indicators of water pollution. During loading of sewage sludge, they can be released into the air reaching 5 × 10^2^ CFU/m^3^. However, to date, there is no data on the complex exposure of WWTP workers to anaerobic bacteria. With this in mind, the aim of this preliminary study was to assess such exposure, taking into account the source of the bacteria in sewage and sludge, as well as the air at workplaces. The analysis also included seasonal variations and size distribution of anaerobic bioaerosol particles.

## Materials and methods

### Sampling sites

The study was carried out in a large (2) wastewater treatment plant in Poland, where the throughput reaches the level of 200,000 m^3^/day. This is a typical mechanical–biological WWTP where anaerobic conditions are a part of the treatment processes. The biological wastewater treatment stage is carried out using activated sludge, in three phases: anaerobic, hypoxic and aerobic. During the treatment process, sewage sludge is produced at a rate of about 250 tons/day, and screenings of about 6 tons/day. Thickened sludge is stabilized by methane fermentation, which is ultimately neutralized by the combustion process. Screenings and sand, after hygienization with chlorinated lime are stored using lagoons. The studied WWTP employs about 150 people.

Samples of sewage and sludge (S1–S6) were collected during the summer season in 6 sampling points in the WWTP, as described in Table 1. Bioaerosol samples (A1–A11) were collected in single repetition in July 2014 and February 2015, at ten workplaces covering different stages of the technological process (Table 2). Additionally, approximately 300 m outside from the plant, the background samples (to relativize the obtained results) were collected. In total, 22 air samples were collected.

**Table 1:** The concentrations of anaerobic bacteria in sewage and sludge samples

Sampling point symbol	Name of sampling point	Concentration		
			GM^a^	GSD^b^
Sewage [× 10^4^ CFU/mL]				
S1	Raw sewage (before the bar screens)	177.5	5.49	85.4
S2	Sewage from primary settling tank	50.7		
S3	Leachate from fermented sludge	0.018		
ANOVA			*p* < 0.001	
Sewage sludge [× 10^4^ CFU/g]				
S4	Screens	425.0	142.7	5.1
S5	Sand from grit remover	390.0		
S6	Dry sludge transported into incineration	17.6		
ANOVA			*p* < 0.001	

^a^Geometric mean

^b^Geometric standard deviation

**Table 2:** The characteristics of sampling point selected for the assessment of airborne anaerobic bacteria in a wastewater treatment plant

Sampling point symbol	Name of sampling point	Temperature (°C)				Relative humidity (%)			
		July		February		July		February	
		GM^a^	GSD^b^	GM	GSD	GM	GSD	GM	GSD
A1	Bar screens	24.8	1.01	12.7	1.04	57.5	1.01	63.9	1.09
A2	Containers with solids in the screens’ hall	24.2	1.01	13.2	1.01	58.0	1.02	63.9	1.09
A3	Primary settling tank—entrance to the control room	24.5	1.04	6.1	1.14	58.0	1.00	73.4	1.07
A4	Sewage sludge pumping station	24.1	1.01	14.5	1.01	58.0	1.05	40.0	1.04
A5	Aeration basins	30.9	1.06	6.7	1.02	39.9	1.11	67.0	1.00
A6	Incineration plant—sludge chute	23.0	1.00	9.3	1.01	61.0	1.02	67.0	1.00
A7	Incineration plant—control room	23.8	1.00	21.8	1.00	52.5	1.04	43.5	1.05
A8	Sludge-thickening building—press and compactors	24.8	1.00	14.3	1.04	67.5	1.03	59.5	1.01
A9	Sludge-thickening building—conveyor belts	24.5	1.00	7.1	1.05	59.5	1.01	69.9	1.08
A10	Sludge-thickening building—control room	24.5	1.01	21.8	1.00	56.0	1.05	40.5	1.02
A11	Background	29.2	1.04	7.2	1.03	40.8	1.15	59.4	1.06

^a^Geometric mean

^b^Geometric standard deviation

### Sampling methods

Sewage and sludge samples were taken directly into 50 mL sterile, screwed-off Falcon tubes and transported to a laboratory for further analysis.

Air samples were stationary collected using 6-stage Andersen impactor (model 10-710, Graseby-Andersen, Inc., Smyrna, USA), which can separate particles of the following aerodynamic diameters: > 7/4.7/3.3/2.1/1.1/0.65 µm. The impactor was set at a height of approx. 0.5 m above the floor or the ground. The sampling time was 5 min, a flow rate of the air was 28.3 L/min and the volume of each collected air sample was 0.1415 m^3^. Calibration of the flow rate was carried out before and after each measurement using a digital flow meter (model Gilibrator-2, Sensidyne, Inc., Clearwater, USA). Between the sampling sessions, an impactor was subjected to disinfection and cleaning with isopropyl alcohol. For sampling of bacterial aerosols, impactor was loaded with Petri plates containing Schaedler agar with 5% additive of sheep blood (bioMérieux, Marcy l’Etoile, France). The graph including size distribution results was created with Microsoft Excel 2010 software (Microsoft Corp., Redmond, USA).

Simultaneously with bioaerosol measurements, at each sampling point, the temperature and relative humidity were measured with the use of portable thermo-hygrometer (model TFA 30.5024, Conrad Electronic GmbH, Hirschau, Germany).

### Laboratory analysis of samples

Sewage and sludge samples were subjected to extraction in saline solution. From these suspensions, three subsequent ten-fold dilutions were made, which were then plated in 1 mL volumes on Schaedler agar with 5% additive of sheep blood (bioMérieux). Plates with sewage and sludge samples together with these from 6-stage Andersen impactor were incubated using AnaeroGen™ system (Oxoid Ltd., Basingstoke, Great Britain) under the following conditions: 2 days (37 °C) + 2 days (30 °C) to allow development of a wide spectrum of bacterial strains with pathogenic properties (Lagier et al. 2015). The final bacterial concentration was expressed in colony-forming units (CFU) present in 1 millilitre of sewage (CFU/mL), 1 gram of sludge (CFU/g) or 1 cubic metre of sampled air (CFU/m^3^). Limit of detection (LOD) of air sampling was 7 CFU/m^3^. Concentration values below LOD (*n* = 3, 13% of samples) were substituted by the lowest determined value divided by the square root of 2.

Microorganisms isolated from plates were identified to genus and/or species level. The analysis of anaerobic bacteria was based on their ability for enzymatic degradation of organic substrates and subsequent detection of the appropriate metabolites generated by these reactions. For this purpose, a biochemical API 20A test (bioMérieux) allowing identification of the clinically important strains was applied.

### Molecular confirmation of *Clostridium* isolates

Taking into account the biochemical imperfections of bacterial identification methods, molecular analysis of *Clostridium* pathogens was also carried out on the basis of 16S rRNA gene sequence analysis. DNA was isolated from pure bacterial cultures using the Genomic Mini Kit (A&A Biotechnology, Gdynia, Poland). The isolation was preceded by incubation with lysozyme (25 µL, 10 mg/mL) to facilitate digestion of bacterial cell walls. Isolated DNA was used as a template for PCR reaction with primer pair specific to bacteria of the genus *Clostridium*: Chis150f (5′-AAAGGRAGATTAATACCGCATAA-3′) and ClostIr (5′-TTCTTCCTAATCTCTACGCA-3′) (Hung et al. 2008) which allow amplification of the gene fragment encoding 16S rRNA. The reaction mixture (20 µL) contained 2 µL of 10 × reaction buffer with MgCl_2_, 0.5 U of RUN-HS Taq Polymerase (A&A Biotechnology), 250 µM of each deoxynucleotide (dNTP), 0.5 µL of each primer (10 pmol/µL) and 0.5 µL of template DNA. Amplification included 35 cycles preceded by initial denaturation (95 °C, 5 min). Each cycle included denaturation (95 °C, 15 s), annealing (58 °C, 60 s) and elongation (72 °C, 60 s) steps. The reaction ended with a final elongation (72 °C, 5 min). The size of the PCR product and the specificity of the primers were checked by performing electrophoretic analysis in 1.5% agarose gel (Certified™ Molecular Biology Agarose, BioRad, Hercules, USA) and comparing the product size to the DNA fragment marker (GeneRuler 1 kb DNA Ladder, Thermo Scientific, Waltham, USA). The photo of PCR products was taken with GelDoc XR+ camera and created in Image Lab Software version 5.2 (BioRad). The reaction product was enzymatically purified using the EPPiC kit (A&A Biotechnology) and sequenced using the Sanger method on the ABI3730 Genetic Analyzer sequencer (Applied Biosystems Inc., Foster City, USA) in the Laboratory of DNA Sequencing and Oligonucleotide Synthesis at Institute of Biochemistry and Biophysics of Polish Academy of Sciences in Warsaw. The resulting sequences were compared to GeneBank nucleotide sequence database (National Center for Biotechnology Information, US National Library of Medicine, USA) using the BLAST (Basic Local Alignment Search Tool) algorithm.

### Statistical analysis

The raw data were used to calculate the geometric means (GM) and geometric standard deviations (GSD). To use the Student’s *t* test, ANOVA and Pearson’s correlation analyses, all data were subsequently log-transformed. To assess taxonomic diversity between the workplace and background samples, the Chi-square (*χ*^2^) test was applied. All calculations were performed using the STATISTICA data analysis software package, version 10. (StatSoft, Inc., Tulsa, USA, 2006), assuming *p* < 0.05 as statistically significant value.

## Results

The average concentration of anaerobic bacteria in the wastewater samples was 5.49 × 10^4^ CFU/mL (GSD = 85.4). The highest values were noted in raw sewage flowing into the treatment plant (P1—1.77 × 10^6^ CFU/mL), and the lowest in the leachate from the digested sludge (P3—185 CFU/mL). Analysis of variance (ANOVA) showed significant differences in concentrations between raw sewage and its subsequent treatment stages (*p* < 0.001). Taking into account anaerobic bacteria in sludge, the average concentration was 1.42 × 10^6^ CFU/g (GSD = 5.1). The most contaminated were the screenings (P4—4.25 × 10^6^ CFU/g), while the least—dry sediment transported to the incinerator (P6—1.76 × 10^5^ CFU/g) and these differences were significant (*p* < 0.001) (Table 1).

The results of the quantitative analysis of airborne bacterial biota are presented in Table 3. The geometric mean concentration of bacterial aerosol in summer was 34 CFU/m^3^, ranging from below the limit of detection (at conveyor belts) to 184 CFU/m^3^ (at containers with the solids in the screens’ hall). The highest average concentration of this aerosol was noted near the aeration basins (127 CFU/m^3^). During the winter series of measurements, a slightly higher geometric mean concentration (75 CFU/m^3^) was observed. The highest winter concentrations of airborne bacteria were found near the bar screens (4.06 × 10^3^ CFU/m^3^) and close to the containers with the solids (1.12 × 10^3^ CFU/m^3^). As in the summer, there was no growth of anaerobic bacteria at the conveyor belts, as well as in the control room in the building of sludge thickening. Statistical analysis revealed no difference between the studied seasons (*t* test *p* > 0.05). On the other hand, the analysis of variance showed significant differences in bacterial concentrations between the studied workplaces, especially between mechanical wastewater treatment processes and sludge thickening (Scheffe test *p* < 0.05 for S1–S3 v. S4, S8–S10). It was also found that the concentrations of bioaerosol at workplaces did not differ significantly from their background level (Scheffe test *p* > 0.05) and they were not significantly determined by the microclimate conditions (Pearson correlation *p* > 0.05).

**Table 3:** The concentrations of anaerobic bacteria in the air at workplaces in the wastewater treatment plant

Technological process (sampling point symbols)	July			February			TOTAL		
	GM^a^ (CFU/m^3^)	GSD^b^	Range (min–max)^c^	GM (CFU/m^3^)	GSD	Range (min–max)	GM (CFU/m^3^)	GSD	Range (min–max)
Mechanical treatment (A1–A3)	77	3.16	21–184	765	6.59	99–4057	243	6.54	21–4057
Sewage sludge treatment (A4, A8–A10)	16	3.35	0–64	16	4.79	0–134	16	3.65	0–134
Biological treatment (A5)	127	–	–	21	–	–	52	3.55	–
Sludge incineration (A6, A7)	22	1.91	14–35	89	1.91	57–141	44	2.62	14–141
Total	34	3.45	0–184	75	8.69	0–4057	50	5.83	0–4057
Background (A11)	49	–	–	7	–	–	19	3.96	–

^a^Geometric mean

^b^Geometric standard deviation

^c^*Min*. minimal value, *max* maximal value

The qualitative analysis of sewage and sludge samples showed the presence of 12 bacterial species belonging to 5 genera: *Actinomyces, Bifidobacterium, Clostridium, Propionibacterium* and *Staphylococcus*. In the sewage sludge, among isolated species, *Clostridium perfringens* was identified. Qualitative analysis of bioaerosol showed the presence of 16 bacterial species belonging to 8 genera (Table 4). It was found that all 16 species were solely identified in the air at mechanical wastewater treatment workplaces (bar screens, containers with solids, primary settling tank). Taxonomical diversity within the abovementioned workplaces was significantly higher compared to background samples (*χ*^2^ = 11.8, *p* < 0.001), the biological treatment stage (*χ*^2^ = 5.1, *p* < 0.05), and the incineration of sewage sludge (*χ*^2^ = 6.6, *p* < 0.05). However, no differences in qualitative composition of bacterial biota were found between sewage and sludge and the air at the workplaces (*χ*^2^ = 0.96, *p* > 0.05). Qualitative analysis of air samples also showed that some of the identified species, such as *Actinomyces meyeri, Bifidobacterium* spp., *Clostridium perfringens* or *Peptostreptococcus* spp., occurred across the whole treatment plant. In turn, the species of the genera *Propionibacterium, Bacterioides* or *Fusobacterium* were characteristic for the primary treatment stages only.

**Table 4:** Qualitative characteristics of anaerobic bacteria present in the wastewater treatment plant samples

Genus/species	Sewage and sludge		Air					Risk group^a^
	Sewage (S1–S3)	Sewage sludge (S4–S6)	Mechanical treatment (A1–A3)	Sewage sludge treatment (A4, A8–A10)	Biological treatment (A5)	Sludge incineration (A6, A7)	Background (A11)	
*Actinomyces* spp.	+	+	+	+	+			2
*Actinomyces israelii*	+	+	+	+	+		+	2
*Actinomyces meyeri*			+	+	+	+		2
*Actinomyces naeslundii*			+	+	+			2
*Bacteroides* spp.			+					1
*Bacteroides distasonis*			+			+		1
*Bacteroides ovatus*			+					1
*Bifidobacterium* spp.	+	+	+	+	+	+	+	1
*Clostridium* spp.	+	+						2
*Clostridium beijerinckii*		+	+	+	+		+	2
*Clostridium botulinum*		+						2
*Clostridium innocuum*			+	+	+	+	+	2
*Clostridium perfringens*		+	+	+	+	+	+	2
*Eggerthella lenta*			+	+	+	+		1
*Fusobacterium mortiferum*			+			+		1
*Peptostreptococcus* spp.			+	+	+	+	+	1
*Propionibacterium acnes*		+						1
*Propionibacterium granulosum*	+							1
*Propionibacterium propionicus*	+		+					1
*Propionibacterium* spp.	+	+	+	+		+		1
*Staphylococcus saccharolyticus*		+						1

^a^According to the Directive 2000/54/EC

**Fig. 1:**
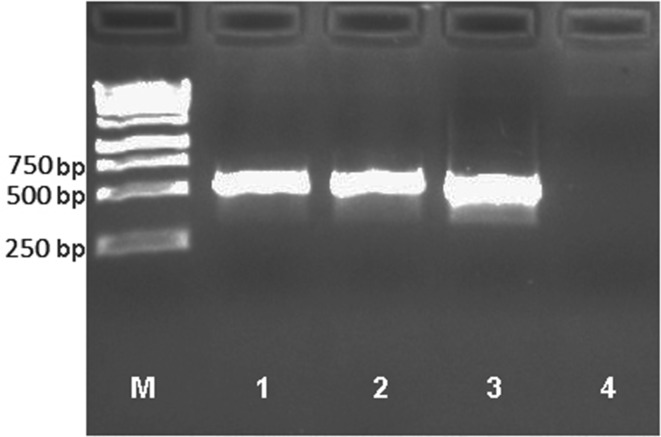
Electrophoretic analysis of PCR product using primers specific to the genus *Clostridium* (Chis150f/ClostIr). As the matrix, DNA isolated from pure cultures of specific strains (identified based on API 20A test) was used 1, *Clostridium beijerinckii*; 2, *Clostridium botulinum*; 3, *Clostridium perfringens*; 4, negative control (instead of DNA, 1 µl of sterile deionised water was added); M, DNA marker

Molecular analysis confirmed the presence of *Clostridium* strains in the wastewater and in the air (Fig. 1); however, DNA sequencing allowed to determine one species, i.e., *Clostridium perfringens* only (16S rRNA sequence similarity 99%). This sequence data were submitted to GenBank with MF444962 accession number.

**Fig. 2:**
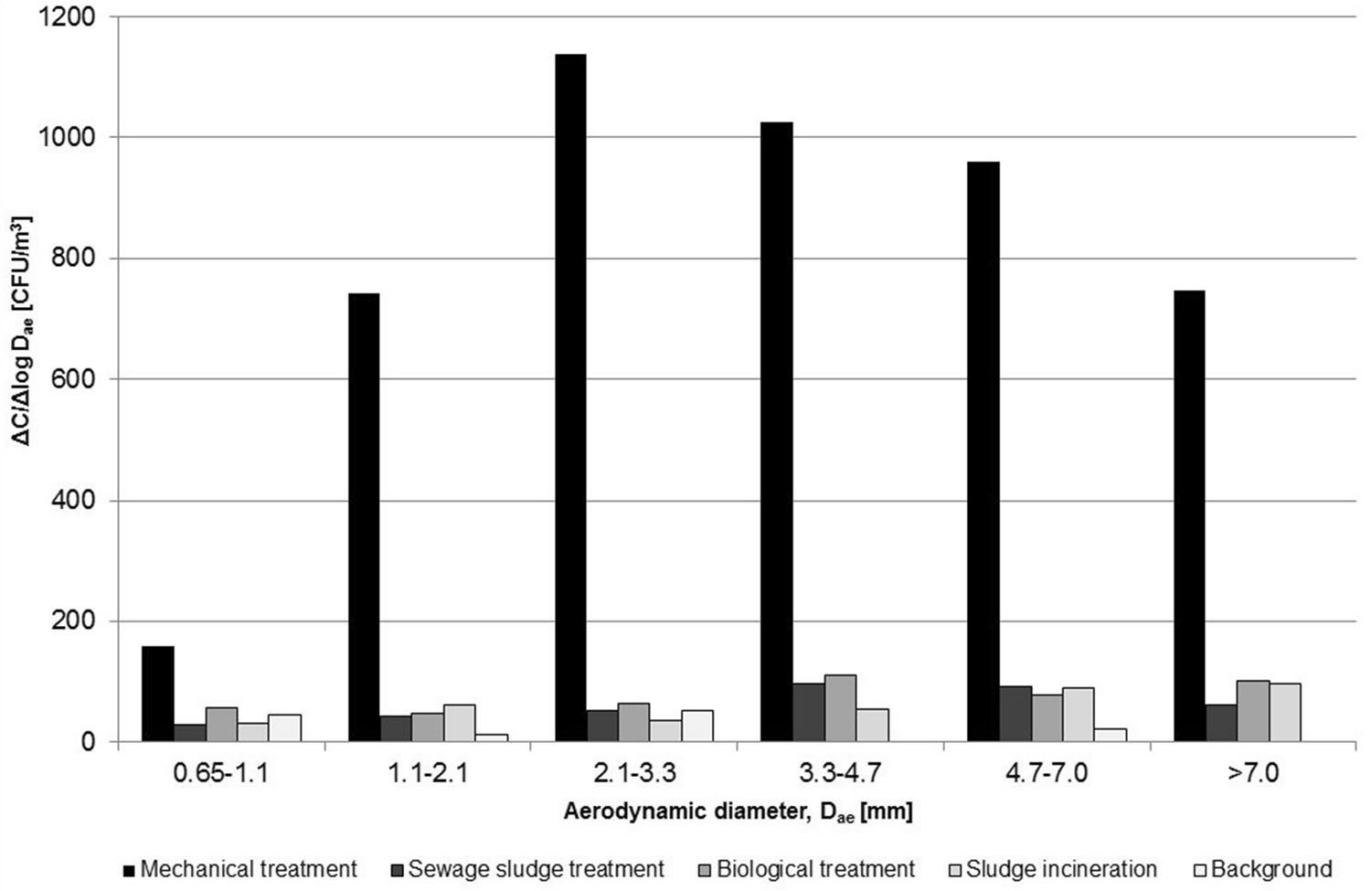
Size distribution of anaerobic bacteria at workplaces in the wastewater treatment plant

Based on the data obtained using the Andersen impactor, it was possible to analyse the size distribution of anaerobic bacteria (Fig. 2). ANOVA analysis showed statistically significant differences between the technological stages of the plant in the whole range of aerodynamic diameters of bacterial aerosol. The bacterial concentrations at mechanical treatment workplaces were significantly higher than those observed during (other) sewage sludge treatment phases (Scheffe test *p* < 0.05). Such differences were probably influenced by bacteria from the genus *Propionibacterium*, which at this sewage treatment stages consisted of about 40% of all the detected microbiota. Analysis of size distribution together with qualitative assessment of isolated species revealed that particles with aerodynamic diameters between 0.65 and 2.1 µm consist of the species of *Eggerthella* and *Bifidobacterium* genera, and between 3.3 and 7 µm—the species of *Bacteroides* and *Actinomyces* genera. Size distribution analysis showed also that above the aerodynamic diameter of 1.1 µm, the process of aggregation of bacterial cells with dust particles and/or sewage droplets was very pronounced.

## Discussion

The present study confirmed that anaerobic bacteria are commonly present in the wastewater treatment plant and the sewage entering the plant is their main source. The sewer environment creates conditions, which favour the growth of anaerobic bacteria. They are involved in different fermentation processes leading to hydrogen sulfide and methane production as well as the release of volatile organic compounds (odours). Among the various anaerobic microorganisms, the most often present are sulfate-reducing bacteria from, e.g., *Desulfovibrio, Desulfotamaculum, Desulfobacter, Desulfuromonas* and *Desulfococcus* genera (Hvitved-Jacobsen 2002; Liu et al. 2015a). It has been also confirmed that bacterial stains from *Simplicispira, Comamonas, Azonexus, Thauera* and other genera are able to form a biofilm on the walls of the sewers (Satoh et al. 2009; Auguet et al. 2015). Hence, the high dynamics of the processes taking place in WWTP environment as well as the variability of physico-chemical conditions may result in a considerable diversity of the microbial communities in the sewage itself (Liu et al. 2015b). Due to that, it is difficult to directly compare how the results of such research perform under different environmental conditions (e.g., in different climate zones and/or seasons).

As soon as the wastewater flows out from the sewers and subsequently become subject to mechanical treatment processes in WWTP, anaerobic bacteria may be easily released from sewage into the air. Such a situation seems to be natural as the first places of sewage purification (such as bar screens, containers with solids and primary settling tanks) are located at the end of sewerage network. In our study, the phenomenon of such emission was confirmed by the highest bacterial concentrations in the wastewater entering the treatment plant, in screenings, in sand from the grit remover and in the air at workplaces. However, as intensive aeration has negative effect on anaerobic bacteria, they were detected at lower levels in the air and water at subsequent treatment stages.

The qualitative analysis showed a great similarity between the bacteria identified in wastewater and in the air, especially regarding *Actinomyces, Bifidobacterium, Clostridium* and *Propionibacterium* genera. In addition, the workplaces where initial phase of wastewater treatment took place were characterized by the largest spectrum of isolated species compared to other sections of the treatment plant. Hence, such a high release rate of bacterial aerosol could be also affected by the location of bar screens and containers with the solids in closed spaces with a limited supply (especially in winter) of the atmospheric air. Similar results were obtained by Fracchia et al. (2006) in the two wastewater treatment plants where the highest microbial concentrations in the air were observed in winter, in the intake chamber (761 CFU/m^3^) and near the primary settling tank (over 4 × 10^4^ CFU/m^3^). Furthermore, during summer, they found that significant number of the analysed samples were below the detection level.

At first glance, the search for anaerobic bacteria in the air seems to be a paradox. In such a case, oxygen should normally limit their number in the airborne state. Nevertheless, research has shown that anaerobic bacteria are able to tolerate oxygen in their environment for a relatively long period of time ranging from 45 min (e.g., *Peptostreptococcus* spp.) up to even 72 h (e.g., *C. perfringens*) (Rolfe et al. 1978). It was also found that in bioreactors, a special ecosystem can be formed in which both aerobic and anaerobic bacteria may coexist contributing in this way to a more efficient wastewater treatment process (Kato et al. 1997).

The qualitative analysis of anaerobic bacteria in the present study showed moderate taxonomic diversity. The presence of some species from *Propionibacterium, Bacteroides, Peptostreptococcus, Bifidobacterium* or *Clostridium* genera seems to have faecal nature and may be directly related to the municipal wastewater. According to some researchers (Ballesté and Blanch 2011; Wéry et al. 2010), these bacteria can be considered as very good indicators of faecal contamination of surface waters. In the present study from among 16 identified species, 7 of them are classified to risk group 2 according to the Directive 2000/54/EC (Directive, 2000) and should be treated as potentially harmful to the health of exposed workers. It should be noted, that the infective dose for most bacteria (including enteric ones) is usually greater than 10^4^ viable cells. However, for some pathogens, even few bacterial cells are needed to cause infection when deposited in the respiratory or digestive systems of a susceptible individual (Crook and Olenchock 1995; Gerardi and Zimmerman 2005).

Among identified bacteria, two species i.e., *Actinomyces israelii* and *Clostridium perfringens* deserve special attention due to their isolation frequency and clinical importance. This actinomycete strain may cause suppurative infections of oral as well as thoracic and abdominal cavities (Mabeza and Macfarlane 2003; Smego and Foglia 1998). In turn, *Clostridium perfringens* is the main etiological agent of myonecreosis of connective tissues (Stevens and Bryant 2002). This bacterium can produce many toxins (including enterotoxin) and enzymes, enhancing in that way its invasiveness. It may be also responsible for food poisoning and diarrhoea (Kądzielska et al. 2012). The growing importance of this species as an indicator of environmental contamination was also confirmed by the fact that in 2008 a draft of the European standard describing the procedure for its determination in sludge, soil and treated bio-waste was prepared (ECN 2008).

Due to the preliminary nature of this study, the qualitative assessment of the isolated strains was mainly based on the biochemical API 20A test, which was preceded with cultivation of bacterial colonies on Schaedler agar. This culture medium guarantees a good growth of anaerobic bacteria, including faecal ones and enables precise selection of colonies for detailed species identification (Murray 1978; Starr et al. 1971). The accuracy of the results obtained using this method are estimated to be 70–85% compared to the other analytical techniques based either on biochemical reactions (Karachewski et al. 1985; Mueller-Spitz et al. 2010) or molecular techniques (Ko et al. 2007). It is clear that molecular techniques utilizing the analysis of 16S rRNA would give more accurate results and it was also shown by our analysis of several samples containing *C. perfringens* species. It is known that the use of real-time PCR allows for quantitative analysis of pathogens during the sewage treatment process (Shannon et al. 2007). However, the aim of this study was to identify the problem only and the use of API 20A test which is dedicated to clinically relevant strains seemed to be cheaper analytical alternative to molecular methods and fully sufficient for this purpose.

The size distribution analysis of studied bacterial aerosol indicated an ability of bacterial cells to form aggregates with both wastewater droplets and organic dust particles of sewage sludge origin. This process was the most pronounced for the particles of aerodynamic diameters between 2.1 and 3.3 µm at workplaces where the mechanical treatment of wastewater took place. The other studied stages of technological processes did not create such substantial aerosolization of bacterial particles. Although there is no similar data for anaerobic bacteria in the scientific literature, the results for aerobic bacteria in the wastewater treatment plant seem to confirm that, in this range of aerodynamic diameters, the concentration of microorganisms in the air is the highest (Li et al. 2013). As the majority of them can be deposited in the lower parts of the respiratory tract, mainly in secondary bronchi, they may be responsible for adverse health outcomes manifested mainly in the form of allergic alveolitis.

## Conclusions

Anaerobic bacteria were widely present both in the sewage and in the air at workplaces in the wastewater treatment plant, reaching the highest concentrations in closed spaces. Some of the identified anaerobic bacteria belonged to the risk group 2 according to the EU Directive 2000/54/EC and should be treated as potentially harmful to the health of exposed workers. Anaerobic bacteria may form aggregates with both wastewater droplets and organic dust particles of sewage sludge origin and as such may be responsible for adverse health outcomes in workers. The control of exposure to anaerobic bacteria should be involved into the risk assessment procedures in wastewater treatment plants and the use of *C. perfringens* as an indicator of microbial contamination appears to be fully justified.
